# Interprofessional Medication Adherence Program for Patients With Diabetic Kidney Disease: Protocol for a Randomized Controlled and Qualitative Study (PANDIA-IRIS)

**DOI:** 10.2196/25966

**Published:** 2021-03-19

**Authors:** Carole Bandiera, Jennifer Dotta-Celio, Isabella Locatelli, Dina Nobre, Grégoire Wuerzner, Menno Pruijm, Faiza Lamine, Michel Burnier, Anne Zanchi, Marie Paule Schneider

**Affiliations:** 1 School of Pharmaceutical Sciences University of Geneva Geneva Switzerland; 2 Institute of Pharmaceutical Sciences of Western Switzerland University of Geneva, University of Lausanne Geneva Switzerland; 3 Center for Primary Care and Public Health (Unisanté) University of Lausanne Lausanne Switzerland; 4 Service of Nephrology and Hypertension Department of Medicine Lausanne University Hospital and University of Lausanne Lausanne Switzerland; 5 Service of Endocrinology, Diabetes and Metabolism Department of Medicine Lausanne University Hospital and University of Lausanne Lausanne Switzerland

**Keywords:** medication adherence, patient compliance, diabetes mellitus, diabetes complications, diabetic nephropathies, chronic kidney disease, kidney failure, renal insufficiency, electronic monitoring, interprofessional program

## Abstract

**Background:**

Despite effective treatments, more than 30% of patients with diabetes will present with diabetic kidney disease (DKD) at some point. Patients with DKD are among the most complex as their care is multifactorial and involves different groups of health care providers. Suboptimal adherence to polypharmacy is frequent and contributes to poor outcomes. As self-management is one of the keys to clinical success, structured medication adherence programs are crucial. The PANDIA-IRIS (patients diabétiques et insuffisants rénaux: un programme interdisciplinaire de soutien à l’adhésion thérapeutique) study is based on a routine medication adherence program led by pharmacists.

**Objective:**

The aim of this study is to define the impact of the duration of this medication adherence program on long-term adherence and clinical outcomes in patients with DKD.

**Methods:**

This monocentric adherence program consists of short, repeated motivational interviews focused on patients’ medication behaviors combined with the use of electronic monitors containing patients’ medications. When patients open the electronic monitor cap to take their medication, the date and hour at each opening are registered. In total, 73 patients are randomized as 1:1 in 2 parallel groups; the adherence program will last 6 months in the first group versus 12 months in the second group. After the intervention phases, patients continue using their electronic monitors for a total of 24 months but without receiving feedback. Electronic monitors and pill counts are used to assess medication adherence. Persistence and implementation will be described using Kaplan-Meier curves and generalized estimating equation multimodeling, respectively. Longitudinal adherence will be presented as the product of persistence and implementation and modelized by generalized estimating equation multimodeling. The evolution of the ADVANCE (Action in Diabetes and Vascular disease: Preterax and Diamicron Modified-Release Controlled Evaluation) and UKPDS (United Kingdom Prospective Diabetes Study) clinical scores based on medication adherence will be analyzed with generalized estimating equation multimodeling. Patients’ satisfaction with this study will be assessed through qualitative interviews, which will be transcribed verbatim, coded, and analyzed for the main themes.

**Results:**

This study was approved by the local ethics committee (Vaud, Switzerland) in November 2015. Since then, 2 amendments to the protocol have been approved in June 2017 and October 2019. Patients’ recruitment began in April 2016 and ended in October 2020. This study was introduced to all consecutive eligible patients (n=275). Among them, 73 accepted to participate (26.5%) and 202 (73.5%) refused. Data collection is ongoing and data analysis is planned for 2022.

**Conclusions:**

The PANDIA-IRIS study will provide crucial information about the impact of the medication adherence program on the adherence and clinical outcomes of patients with DKD. Monitoring medication adherence during the postintervention phase is innovative and will shed light on the duration of the intervention on medication adherence.

**Trial Registration:**

Clinicaltrials.gov NCT04190251_PANDIA IRIS; https://clinicaltrials.gov/ct2/show/NCT04190251

**International Registered Report Identifier (IRRID):**

DERR1-10.2196/25966

## Introduction

Currently, approximately 463 million people have been diagnosed with diabetes worldwide [1]. In Switzerland, it is estimated that approximately 500,000 people have diabetes, representing 5.7% of the population [2,3]. Diabetes is associated with a burden of microvascular and macrovascular complications. Diabetic kidney disease (DKD), a microvascular complication of diabetes, affects 30%-40% of the patients with diabetes. Although DKD is the main cause of end-stage renal disease (ESRD), most patients will die of cardiovascular complications before reaching ESRD. Patients with DKD are among the most complex patients receiving diabetes care. Their care is multifactorial and multidisciplinary, involving different groups of health care providers. The multifactorial approach involves pharmacological treatment of various cardiovascular risk factors, including glucose levels, cholesterol levels, and blood pressure levels, and the pharmacological treatment of complications secondary to the reduced renal function. Patients with DKD often take more than 5 daily treatments. Polypharmacy is associated with suboptimal adherence, with risk of an accelerated decline in renal function and ESRD [4]. Although 40% of the patients with DKD do not adhere to their treatments [5,6], literature on medication adherence intervention programs addressing specifically DKD patients is scarce. Recent evaluations showed better metabolic and blood pressure control in patients participating in such programs [7,8]. Furthermore, a high level of self-management, including taking medications, increases the quality of life [6,9]. However, the results of most studies were not conclusive, as medication adherence was evaluated by subjective questionnaires with poor reliability. A feasibility study in patients with DKD who participated in an intervention combining a medication plan and regular phone calls by a specialist nurse did not demonstrate an increase in the medication adherence [10]. Another study concluded that intensive behaviors and interprofessional interventions delayed renal function decline [11]. However, to our knowledge, no study has evaluated the long-term impact of an adherence program or the optimal duration of such a program. It is necessary to better understand the needs of patients with DKD in terms of medication management to achieve better medication adherence and clinical outcomes and slow cardiovascular and renal disease progression.

In 1993, the pharmacy of Unisanté developed an interprofessional medication adherence program (IMAP) to support patients with chronic diseases with their drug management, combining the use of a medication event monitoring system (MEMS and MEMS AS, Aardex Group) and motivational interviews [12]. The first few studies showed encouraging results in improving medication adherence, clinical targets, and retention in care for chronically ill patients [13-18]. The PANDIA-IRIS (patients diabétiques et insuffisants rénaux: un programme interdisciplinaire de soutien à l’adhésion thérapeutique) study is based on this routine medication adherence program led by pharmacists. The global aim of this study is to evaluate the impact of the medication adherence program on long-term adherence and clinical outcomes in patients with DKD.

## Methods

### Ethical Considerations

The PANDIA-IRIS study was approved by the local ethical committee (Vaud, Switzerland) in November 2015. Since then, 2 amendments to the protocol version 4 have been accepted—in June 2017 and October 2019. This study is being carried out in accordance with the protocol and with the principles of the current version of the Declaration of Helsinki. The results of this study will be submitted to local, national, and international conferences and to a peer-reviewed journal for publication. This protocol has been written according to the SPIRIT reporting guidelines [19].

### Objectives

The primary objective of the PANDIA-IRIS study is to assess whether the length of the IMAP (12 vs 6 months) has an impact on medication adherence at 6, 12, 18, and 24 months after enrolment. As a secondary objective, this study will explore whether IMAP intervention changes the ADVANCE (Action in Diabetes and Vascular Disease: Preterax and Diamicron Modified-Release Controlled Evaluation) [20] and UKPDS (United Kingdom Prospective Diabetes Study) [21] clinical scores of patients with type 2 diabetes at 6, 12, 18, and 24 months after enrolment. Another secondary objective is to explore patient satisfaction with the medication adherence program through 30-minute semistructured qualitative interviews between an investigator and a subgroup of 14 patients included in the PANDIA-IRIS study. The main hypothesis is that patients participating in the 12-month adherence program will have better implementation and persistence, a higher long-term medication adherence rate, and better ADVANCE and UKPDS clinical scores at 12, 18, and 24 months after enrolment than patients in the 6-month intervention group.

### Trial Design

**Figure 1:**
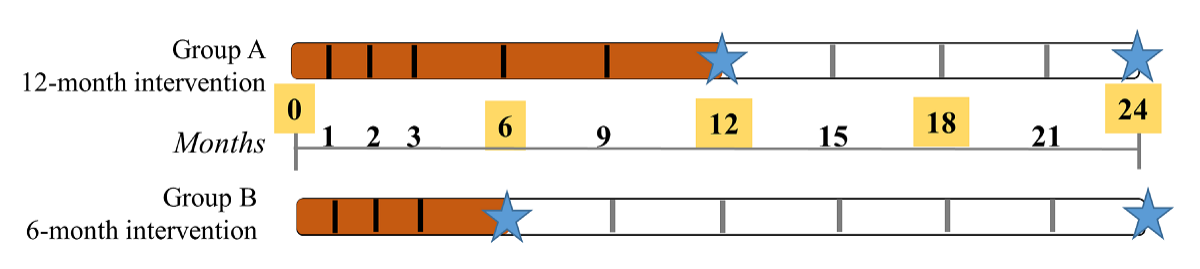
Design of the PANDIA-IRIS (Patients diabétiques et insuffisants rénaux: un programme interdisciplinaire de soutien à l’adhésion thérapeutique) study.

The PANDIA-IRIS design uses a mixed approach as it combines (1) a monocentric, prospective, and open randomized controlled trial and (2) a qualitative study. Patients will be randomized 1:1 in 2 parallel groups; patients of group A will attend the medication adherence program for 12 months and patients randomized to group B will attend the program for 6 months. Satisfaction with this study will be evaluated with selected patients through a qualitative interview at the end of the intervention phase or at the end of this study, or at drop-out (Figure 1). The legend for Figure 1 is available in Multimedia Appendix 1.

### Enrolment of the Participants

This monocentric study is being conducted in the diabetes and kidney outpatient clinics in the University Hospital (CHUV, Lausanne, Switzerland), as well as in the outpatient clinic and the University community pharmacy of the Center for Primary Care and Public Health (Unisanté, Lausanne). The PANDIA-IRIS study is voluntary and each patient had to sign an informed consent form to be enrolled. Recruitment began in April 2016 and ended in October 2020. Eligible patients were identified each week through electronic database screening of all patients with diabetes followed in the University Hospital outpatient clinics. Investigators introduced this study to the eligible patients during a clinical appointment. Recruitment for the qualitative study began in July 2017 and ended in August 2020.

### Randomized Controlled Medication Adherence Study

The inclusion and exclusion criteria are presented in Textbox 1 and Textbox 2, respectively.

**Inclusion criteria for the participants.**

- Adults older than 18 years who are being followed in the University Hospital and have type 2, type 1, latent autoimmune diabetes in adults, or corticoid-induced diabetes
- One of the following biochemical criteria: estimated glomerular filtration rate (eGFR)≤60 mL/min/1.73 sq m, eGFR>60 mL/min/1.73 sq m that has deceased >5 mL/min/year, urine albumin/creatinine ratio >30 mg/mmol, patients hospitalized at least twice for acute renal impairment in the past 5 years.
- Treatment with at least one of the following drug classes: oral antidiabetic drugs (metformin, dipeptidyl peptidase-IV inhibitors, sodium-glucose cotransporter-2 inhibitors, sulfonylureas, glinides), statins, diuretics, beta-blockers, calcium antagonists, alpha-blockers, angiotensin-converting-enzyme inhibitors, angiotensin II receptor blockers, or aspirin.
- Fluency in French or English. If not, the presence of an interpreter at each medication adherence visit.
- Full blood test (eGFR, glycated hemoglobin, albumin/creatinine ratio or total, high density lipoprotein and low-density lipoprotein cholesterol) in the 6 months prior to inclusion.

**Exclusion criteria for the participants.**

- Incapacity to make decisions, having cognitive disorders, or being under tutelage
- Pregnancy
- Active cancer (not in the remission phase)
- Treatment of the patient is managed by nursing homes or home care services
- Patients already included in an intervention study

All the pharmacists providing the IMAP intervention were trained in motivational interviewing at the University community pharmacy of Unisanté [12]. The technicians were trained in how to complete the electronic monitor data, count pills, and use the MedAmigo software (MEMS and MEMS AS, Aardex Group) to upload electronic monitor adherence data and generate a medication adherence report.

### Qualitative Study

Semistructured interviews are conducted with a subgroup of 14 patients included in the PANDIA-IRIS study either at the end of the intervention phase so that the patient remembers the motivational interviews well or at the end of the study so that the patient can discuss the postintervention monitoring phase, or at drop-out. To ensure heterogeneity among the selected patients, patients meeting at least one of the following criteria will be enrolled prospectively: male, female, patients from group A, patients from group B, patients who completed the 24 months of the study, patients who just ended the intervention phase, patients who dropped out, and patients having an adherence rate >95% or <80%.

### Intervention

#### Use of the Electronic Monitor

In both groups, patients use electronic monitors for 24 months. Electronic monitors register the date and hour of each opening, which corresponds to the moment of the drug intake as long as both behaviors are consecutive. Nevertheless, one limitation of this tool is that patients could open the electronic monitor without taking the drug or swallowing it long after the electronic monitor opening. In order to limit this bias and before showing the electronic monitor data to the patient at each pharmacy visit, pharmacists check with the patients that each opening corresponds to the drug intake. They ask the patient about the usual lapse of time between each opening and the real time of the drug intake. The liquid-crystal display (LCD) screen on top of the electronic monitor cap resets at 3 AM each morning. Hence, each time the patient opens the electronic monitor cap, the LCD shows the number of cumulative openings until 3 AM on the next day. The LCD screen reminds the patient about whether the medication has been taken or not.

Drugs to monitor are prioritized based on the following algorithm, which we constructed based on side-effect profiles: (1) all oral antidiabetic drugs, (2) statins, (3) diuretics, (4) beta-blockers, (5) calcium antagonists, (6) alpha-blockers, (7) angiotensin-converting-enzyme inhibitors, (8) angiotensin II receptor blockers, and (9) aspirin. Moreover, in each class, eligible drugs taken more than once a day prioritize monitoring. The maximum number of monitored drugs is 6. Physicians can modify the treatments at any time. If a patient has to stop all the monitored drugs, he or she will stop the study but the data collected until then will be kept for analysis. If a drug is switched to another eligible drug, the new prescribed drug will be monitored.

#### Medication Adherence Intervention

All included patients will benefit from the IMAP in addition to routine clinical care. The frequency of IMAP visits is the same in both groups throughout the 24 months: once per month for the first 3 months, followed by 1 visit every 3 months (Figure 1). Pharmacists lead short, repeated semistructured motivational interviews based on the theoretical Fisher model [22]. First, patients’ knowledge of treatments is evaluated. Then, motivation to take the treatment, self-efficacy, daily medication behaviors, and side-effect management are explored in an empathetic nonjudgmental way. The graph representing electronic monitor daily openings (dates and hours) since the last visit is shown to the patient as feedback. Adherence results are discussed to reinforce acquired adherent behaviors and to understand nonadherent episodes, and then, solutions with tailored elements are constructed to address nonadherence. To ensure interprofessional collaboration, the pharmacist sends a semistructured report after each medication adherence interview to the clinical team, including the endocrinologist, nephrologist, general practitioner, nurse, dietician, and psychologist. During the COVID-19 pandemic, adherence interviews are conducted through phone calls for some patients, if the clinicians considered them at high risk of complications following infection by SARS-CoV-2. If the interview is conducted by phone, it is notified on the case report form.

#### Qualitative Study

At the end of the intervention phase or at the end of this study or at drop-out, patients are invited to express their opinions on the medication adherence program and the overall study. Patients who signed the informed consent form specific to the qualitative part of the PANDIA-IRIS study participate in 30-minute in-depth semistructured interviews. The main themes covered are the evaluation of the quality of the medication adherence interview, the positive and negative aspects of the program, the opinions of the patient regarding the electronic monitor, and if the patients completed this study, the perceived impact of the program during the 12-month to 18-month follow-up period.

### Postintervention Monitoring Phase

After the intervention phase, patients will still use electronic monitors with their LCD screens, but their adherence data will be double-blinded. Patients will not receive any feedback or motivational interviews about their daily drug management. The clinical team will not obtain any adherence reports. Due to ethical reasons, if the medication adherence rate detected by the MedAmigo software is less than 30% for at least one drug for 2 consecutive pharmacy visits, the software will automatically notify the pharmacist, and the patient will stop the study and will be oriented to the routine IMAP. If a physician in charge of a patient explicitly asks to access the adherence data during the postintervention monitoring phase for clinically urgent and important reasons, the principal investigator will sign a permission form that will be included in the case report form, and the patient will stop the study. All the data collected until then will be kept for analysis. At 18 months after the inclusion, during the postintervention monitoring phase, blood and urine samples will be collected to determine the clinical values at this time point. Indeed, the frequency of blood and urine samples collection differs between patients, and collecting laboratory values for each patient at this precise time point will ensure consistency in the analysis.

### Assignment of Interventions

Patients are randomized 1:1 at inclusion to either the 12-month or 6-month intervention group. According to the risk of nonadherence due to the adverse effects of statins or the complexity of the drug regimen, 4 randomization groups were created: patients treated with statins, patients who have to take at least one drug more than once daily, patients with both of the former conditions, and patients with none of the former conditions. Then, the randomization is performed in each strata and is coded with 0, corresponding to group A, where patients receive the intervention for 12 months, or 1, corresponding to group B, where patients receive the intervention for 6 months. The randomization code is generated by a random repetition of 4 and 6 blocks composed of 1 or 0 (Excel, Microsoft) to prevent predictability of the sequence. The randomization sheets were created by an independent researcher from the Unisanté Research Support Unit. The allocation is established for each consecutive patient by unsealing the randomization sheet. This study is open, and the clinical team as well as the patients are aware of the allocation group.

### Outcomes

#### Primary Outcomes

The global adherence outcome is defined as the percentage of days a patient correctly takes the medication across the individual study duration. The mean global adherence will be compared between groups. According to the EMERGE (ESPACOMP Medication Adherence Reporting Guidelines), medication behaviors will also be described longitudinally in both groups through 3 operational definitions: implementation, persistence, and longitudinal adherence at 6, 12, 18, and 24 months after enrolment [23].

#### Secondary Outcomes

The ADVANCE and UKPDS clinical scores will be assessed and compared at baseline, 6, 12, 18, and 24 months after enrolment for patients with type 2 diabetes in both groups. The ADVANCE score indicates the risk of developing cardiac complications associated with type 2 diabetes within 4 years and estimates the risk of developing renal complications within 5 years or more than 5 years. The UKPDS score calculates the risk of fatal coronary heart disease or stroke in patients with type 2 diabetes with no antecedent cardiovascular disease. These scores are calculated on the following parameters: sex, ethnicity, smoking status, time since diabetes diagnosis, blood pressure, glycated hemoglobin, total and high-density lipoprotein cholesterol, atrial fibrillation, albumin in urine, abdominal circumference, and BMI [24].

During the postintervention monitoring phase, the number of patients with less than 30% adherence for at least one drug during 2 consecutive visits as well as the time-to-event will be compared between the groups. We will also describe implementation and persistence at 6 months and 12 months after the end of the intervention in both groups to explore the impact of the length of the intervention on adherence. Patient satisfaction regarding the program will be analyzed through qualitative interviews.

#### Sample Size

An expert committee composed of nephrologists, endocrinologists, nurses, pharmacists, and statisticians agreed that the difference in the mean global adherence between groups should be 5%. The hypothesis states that the adherence rate will average 97.5% in the 12-month intervention group and 92.5% in the 6-month intervention group. A standard deviation of 1.7 was assumed in the logit scale in both groups. This corresponds to 95% global adherence in the intervals (57.5%-99.9%) and (30.0%-99.7%) for the 12-month intervention group and the 6-month intervention group, respectively. According to these parameters, a sample size calculation was made simulating individual series of daily medication (1=at least the correct number of daily openings of the electronic monitor for all drugs monitored; 0=fewer daily openings than prescribed for at least one drug monitored), in order to consider both the interindividual variability (SD 1.7) and the intraindividual variability (the measurement error). Considering that the mean number of measures of a subject will be 365, 72 patients must be included in this study (36 participants in each group) to have a power of 80% and a two-tailed alpha error of .05. Regarding the qualitative study, the expert committee agreed that a subgroup of 14 included patients will attend the qualitative interview but more patients may be included if data saturation is not reached after the completion of 14 interviews.

#### Participant Timeline and Data Collection

Data collection is performed by the investigators and is prospectively registered in the platform Research Electronic Data Capture (RedCap), a secure web app for building databases. Each patient has a unique study number generated by RedCap. First, during recruitment, if eligible patients refuse to participate, their reasons for doing so are recorded. For patients included in this study, their sociodemographic data are provided by the administrative software of the hospital at inclusion. Clinical data for each visit are provided by the patient electronic clinical file (Soarian, Cerner) and reported in the case report form. The participant timeline and data collection process are shown in Multimedia Appendix 2 [25] for both groups. Electronic monitor data are uploaded on the secured MedAmigo web portal at each medication adherence visit during the intervention and at each medication refill during the follow-up for both groups. Concomitantly, the patient’s electronic monitor use is systematically checked by the pharmacist through a set of validation questions (ie, identification of nonmonitored periods, eg, during hospitalizations, whether medication was sometimes prepared in advance for a later use since the last visit). A pill count is performed by the technician during the intervention phase and by a blinded independent researcher during the postintervention monitoring phase. The participants who leave this study before completion are not replaced and are considered as dropouts but the data collected until then will be kept for analysis. The data monitoring committee is composed of the main investigators of the PANDIA-IRIS study. After the end of this study analysis, data will be archived and kept for 10 years in the secure data warehouse of the University Hospital before destruction. The database of the PANDIA-IRIS study will be shared in a secured data repository of the University of Geneva (Yareta) at the end of the analysis.

### Data Analysis

#### Sociodemographic and Clinical Data

Sociodemographic and clinical data will be presented as percentages, means, and standard deviations or medians and interquartile ranges depending on the distribution of the data.

#### Medication Adherence

All dates and the timing of electronic monitor activation will be used to analyze medication adherence. Electronic monitor data will be reconciled with pill counts and patient reports [26]. The global adherence will be calculated as the percentage of days during which the drug is taken properly across the individual study duration. Two-sided Student *t* test will be used to compare the mean probabilities between groups in the logit scale. A difference between groups will be considered statistically significant if *P*<.05. Medication behaviors are characterized by implementation, persistence, and longitudinal adherence [27].

Implementation evaluates at each day the proportion of patients taking at least the correct dose prescribed on that day among the patients who are still participating in the program. For each participant, the daily medication behaviors will be described with a binary variable (1=at least the correct number of daily openings of the electronic monitor for all drugs monitored; 0=fewer daily openings than prescribed for at least one drug monitored). This code is commonly used and cannot discriminate overadherence, which will be considered as adherence. Implementation will be modeled with the generalized estimating equation multimodeling [28].

Persistence corresponds to the distribution of times between inclusion in the study and discontinuation (ie, unilateral stopping of treatment by the patient). Persistence will be described with a Kaplan-Meier survival curve. The log rank test will be used to compare the persistence between groups [28].

Longitudinal adherence is defined at each day as the proportion of patients taking at least the correct dose prescribed on that day among all the patients included in the study. Longitudinal adherence will be presented as the product of persistence and implementation and modelized by generalized estimating equation multimodeling [28]. In an exploratory analysis, univariable and multivariable regressions will analyze the associations among medication behaviors (combining persistence and implementation), sociodemographic and clinical data at inclusion, and changes in the clinical and biochemical variables at the completion of the study.

The number of patients with adherence rates less than 30% in the postintervention monitoring phase for at least one drug during 2 consecutive pharmacy visits will be compared between groups with a chi-squared test or with Fisher exact test if the sample size is less than 5 patients. The statistician who will perform the analysis will not be aware of the group allocation until the data are completely frozen. Statistical analysis will be performed with the R statistical package (version 3.6, The R Foundation for Statistical Computing) [29].

#### ADVANCE and UKPDS Clinical Scores

The changes in the ADVANCE and UKPDS scores during the 24-month study will be analyzed with generalized estimating equation multimodeling, considering collected covariables such as sociodemographic data, monitored treatments, and medication adherence.

#### Qualitative Study

Audio recordings will be transcribed verbatim, and a content analysis will be performed using MAXQDA (VERBI GmbH) software. The codes will be labeled and categorized into themes. To ensure the codification validity, 2 investigators will codify the verbatim transcription independently and discrepancies will be discussed.

#### Missing Data

Missing data will be clearly identified in the results tables and will not be computerized.

### Patient and Public Involvement

Patients or the public were not involved in the design, conduct, reporting, or dissemination plans of our research. Once the results of the PANDIA-IRIS study will be published, the included patients will be informed about the results through a summary flyer using lay language.

## Results

Patients’ recruitment began in April 2016, ended in October 2020, and it was a challenging task. Indeed, this study was introduced to all consecutive eligible patients (n=275). In total, 73 out of 275 patients accepted to participate (26.5%) and 202 (73.5%) patients refused. Most patients who refused to participate argued that they had developed satisfactory skills and habits in drug self-management over time and that this study would disturb their routine. For instance, most patients already used a weekly pill organizer and were not ready to replace it with electronic monitors. Nevertheless, investigators could not confirm that these patients had no adherence issue. A new quantitative and qualitative substudy has been planned to understand the sociodemographic and clinical variables that differ between the patients who accepted versus those who refused to participate. Qualitative interviews will be conducted with a representative number of patients who refused to participate to understand their personal reasons for nonparticipation in order to further improve the routine IMAP. Patient recruitment is completed, and sociodemographic and clinical data are prospectively collected for the included patients until October 2022. Data analysis will be performed and results will be submitted for publication in a peer-reviewed open access journal.

## Discussion

### Strengths of This Study

The PANDIA-IRIS study is the first study to analyze the impact of a pharmacist-led adherence program on long-term medication adherence and cardiovascular scores in patients with diabetes and renal impairment. This study is based on a robust methodology; patients use electronic monitors, often considered as close to the gold standard to assess medication adherence. Moreover, patients are included in the routine IMAP that already led to significant improvements in the medication adherence and clinical outcomes of other chronically ill patients [12,17,18]. The mixed methodology is another strength of this study; the qualitative satisfaction interviews led with a representative number of included patients will enable the research and pharmacy teams together to further adapt the program to the specific needs of patients having DKD.

### Limitations

This study has some limitations. First, patients are included for 24 months in this study, which is a relatively long period of time, thereby increasing the probability of change in regimen over time and dropouts. In addition, the adherence data analysis is complex as patients use more than 1 electronic monitor. However, complex medication adherence data analysis was previously developed by the investigators, and the statistician who will perform the analysis is an expert in this field. Secondly, we cannot exclude the Hawthorne effect during the first 6 weeks of this study, explained by the fact that participants might improve their usual medication adherence behaviors as they feel observed [30]. We will analyze adherence as a trajectory (longitudinal, repeated measures) and we will pay attention to this risk during our analysis. All limitations and their possible impacts on the results will be clearly depicted in the final publication.

### Conclusion

The PANDIA-IRIS study will provide crucial information about the impact of the medication adherence program on adherence and the clinical outcomes of the patients with DKD who are known for polypharmacy challenges and who are engaged in complex and multifactorial care. Monitoring medication adherence during the postintervention phase is innovative in this field. This analysis will help health care providers to better support medication adherence in patients with DKD.

## Appendix

Multimedia Appendix 1 Caption of the design of the PANDIA-IRIS study.

Multimedia Appendix 2 Schedule of enrollment, interventions, and data collection for the PANDIA-IRIS study.

Multimedia Appendix 3 CONSORT-eHEALTH checklist (V1.6.1).

## Abbreviations

ADVANCE: Action in Diabetes and Vascular Disease: Preterax and Diamicron Modified-Release Controlled Evaluation
DKD: diabetic kidney disease
ESRD: end-stage renal disease
IMAP: interprofessional medication adherence program
LCD: liquid-crystal display
MEMS: medication event monitoring system
PANDIA-IRIS: Patients diabétiques et insuffisants rénaux: un programme interdisciplinaire de soutien à l’adhésion thérapeutique
RedCap: research electronic data capture
UKPDS: United Kingdom Prospective Diabetes Study
