# Supplementary figures and images for: Interprofessional Medication Adherence Program for Patients With Diabetic Kidney Disease: Protocol for a Randomized Controlled and Qualitative Study (PANDIA-IRIS)
Source: JMIR Res Protoc. 2021 Mar 19;10(3):e25966. doi: 10.2196/25966 (PMC8088877; doi:10.2196/25966)

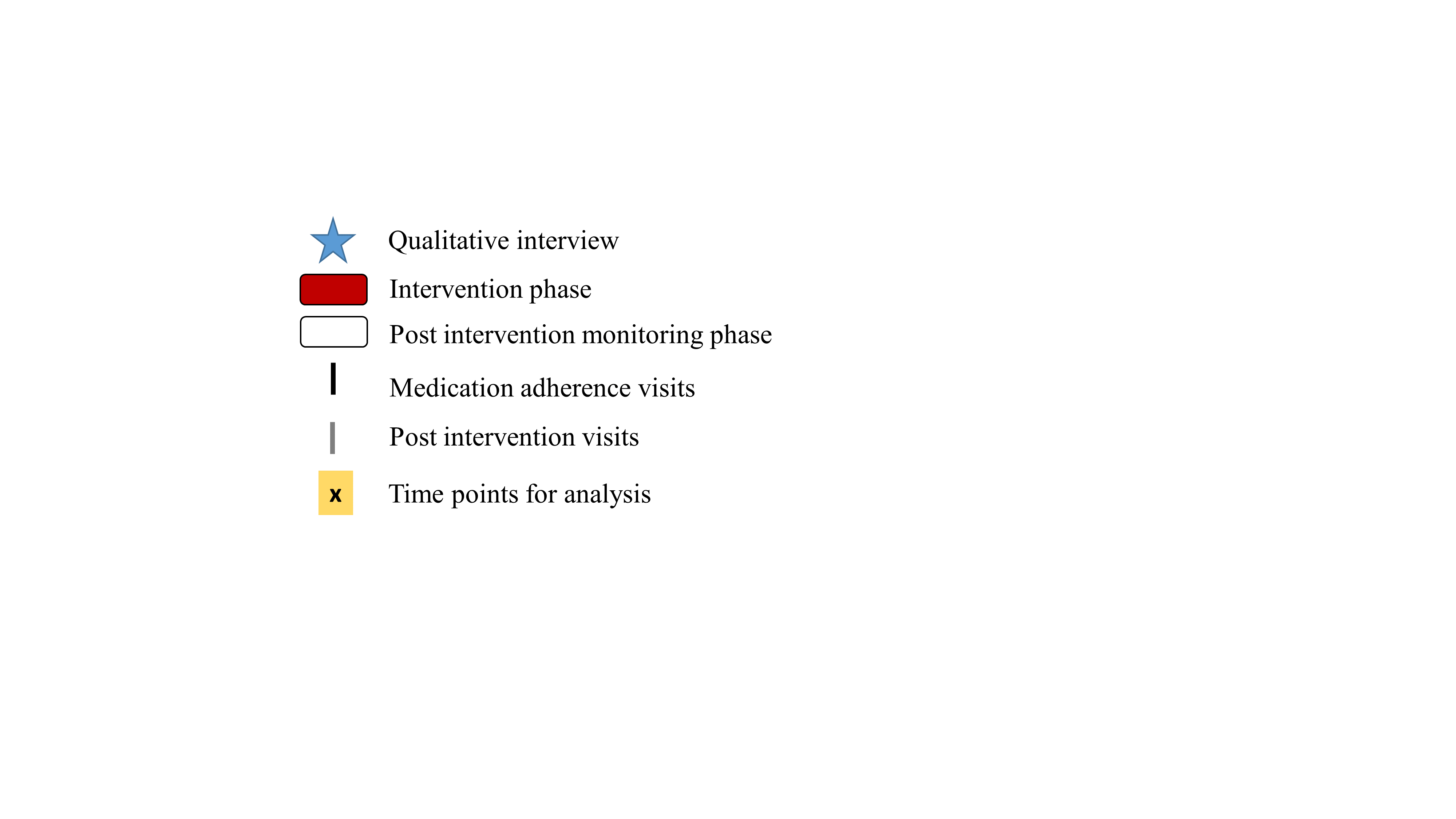

Supplement: Multimedia Appendix 1 [file resprot_v10i3e25966_app1.png]
