# Supplementary material for: Interprofessional Medication Adherence Program for Patients With Diabetic Kidney Disease: Protocol for a Randomized Controlled and Qualitative Study (PANDIA-IRIS)
Source: JMIR Res Protoc. 2021 Mar 19;10(3):e25966. doi: 10.2196/25966 (PMC8088877; doi:10.2196/25966)
Supplement: Multimedia Appendix 2 [file resprot_v10i3e25966_app2.docx]

## Multimedia Appendix 1

**Table S1.** Schedule of enrolment, interventions and data collection for the PANDIA-IRIS study

| **PANDIA-IRIS study period** | | | | | | | | | | | | |
| --- | --- | --- | --- | --- | --- | --- | --- | --- | --- | --- | --- | --- |
| **Description of the event** | **Screening** | **Enrolment** | **Post allocation** | | | | | | | | | **Close-out/end of study** |
| **Time points** | T-1 | T0  [d0] | T1  [d30] | T2  [d60] | T3  [d90] | T4  [d180] | T5  [d270] | T6  [d360] | T7  [d450] | T8  [d540] | T9  [d630] | T10  [d720] |
| **Eligibility screening and recruitment** | ✓ |  |  |  |  |  |  |  |  |  |  |  |
| **Recruitment data collection** | | | | | | | | | | | | |
| **Reasons for patient refusal (descriptive)** | ✓ |  |  |  |  |  |  |  |  |  |  |  |
| **Inclusion of patients** | | | | | | | | | | | | |
| **Informed consent** |  | ✓ |  |  |  |  |  |  |  |  |  |  |
| **Allocation (group A or B^a^)** |  | ✓ |  |  |  |  |  |  |  |  |  |  |
| **Qualitative interview** |  |  |  |  |  | B |  | A |  |  |  | A, B |
| **Medication adherence data collection** | | | | | | | | | | | | |
| **Intervention phase: open reading of adherence EM data, adherence conciliation by pill count and patient report, EM refills and adherence report sent to the clinical team** |  |  | A, B | A, B | A, B | A, B | A | A |  |  |  |  |
| **Post-intervention monitoring phase: double-blind reading of adherence EM data, patient report about EM use, EM refills** |  |  |  |  |  |  | B | B | A, B | A, B | A, B | A, B |
| **Sociodemographic and clinical data collection in both groups** | | | | | | | | | | | | |
| **Sociodemographic data^b^**  **Type of diabetes (1, 2, LADA^c^, other)**  **Time since diabetes diagnosis (years)**  **Mean eGFR decline (ml/min/1.73 m^2^/year)**  **Treated hypertension (yes/no)**  **Retinopathy (yes/no)**  **Arterial fibrillation (yes/no)** |  | ✓ |  |  |  |  |  |  |  |  |  |  |
| **Blood pressure measurement (mmHg)**  **eGFR (ml/min/1.73 m2)**  **Creatinine blood level ([μ](https://fr.wiktionary.org/wiki/%CE%BCmol#conv)mol/L)**  **HbA1c (%)** |  | ✓ | ✓ | ✓ | ✓ | ✓ | ✓ | ✓ | ✓ | ✓ | ✓ | ✓ |
| **Urine albumin (mg/L)**  **Urine creatinine ([μ](https://fr.wiktionary.org/wiki/%CE%BCmol#conv)mol/L)** |  | ✓ |  |  | ✓ | ✓ | ✓ | ✓ | ✓ | ✓ | ✓ | ✓ |
| **Measurement of abdominal circumference (cm)**  **BMI**  **Total, HDL and LDL cholesterol (mmol/L)**  **Triglycerides (mmol/L)** |  | ✓ |  |  |  | ✓ |  | ✓ |  | ✓ |  | ✓ |
| **Smoking status score^d^** |  | A, B |  |  |  | B |  | A |  |  |  | A, B |
| **Treatment data collection** | | | | | | | | | | | | |
| **Each monitored molecule, dose, regimen**  **Co-treatments** |  | ✓ | ✓ | ✓ | ✓ | ✓ | ✓ | ✓ | ✓ | ✓ | ✓ | ✓ |
| **Drop-outs (end of the study before the 24-month completion)** |  |  |  |  |  |  |  |  |  |  |  | ✓ |

### ^a^Group A: intervention delivered for 12 months; Group B: intervention delivered for 6 months

### ^b^Age, sex, civil status, nationality, ethnicity, level of education, age at the end of mandatory schooling, previous use of an adherence support tool or adherence programme

^c^Latent autoimmune diabetes in adults (LADA)
^d^Using the Di-Prochaska Di Clemente validated questionnaire[30]
